# How to Design a Remote Patient Monitoring System? A French Case Study

**DOI:** 10.1186/s12913-020-05293-4

**Published:** 2020-05-19

**Authors:** Marie Ferrua, Etienne Minvielle, Aude Fourcade, Benoît Lalloué, Claude Sicotte, Mario Di Palma, Olivier Mir

**Affiliations:** 1 Capri program, Research Division, Gustave Roussy, Villejuif, France; 2 I3, CRG, Ecole Polytechnique, CNRS, Palaiseau, France; 3 Lorraine University, CNRS, Inria, Nancy, France; 4 EHESP, Department of Health Care Management, Rennes, France; 5 American Hospital, Neuilly-sur-Seine, France; 6 Outpatient Department, Gustave Roussy, Villejuif, France

**Keywords:** Implementation, Remote Patient Monitoring system, Care coordination, Complex intervention, Oncology

## Abstract

**Background:**

Remote Patient Monitoring Systems (RPMS) based on e-health, Nurse Navigators (NNs) and patient engagement can improve patient follow-up and have a positive impact on quality of care (by limiting adverse events) and costs (by reducing readmissions). However, the extent of this impact depends on effective implementation which is often restricted. This is partly due to the lack of attention paid to the RPMS design phase prior to implementation. The content of the RPMS can be carefully designed at this stage and various obstacles anticipated. Our aim was to report on an RPMS design case to provide insights into the methodology required in order to manage this phase.

**Methods:**

This study was carried out at Gustave Roussy, a comprehensive cancer centre, in France. A multidisciplinary team coordinated the CAPRI RPMS design process (2013–2015) that later produced positive outcomes. Data were collected during eight studies conducted according to the Medical Research Council (MRC) framework. This project was approved by the French National Data Protection Authorities.

**Results:**

Based on the study results, the multidisciplinary team defined strategies for resolving obstacles prior to the implementation of CAPRI. Consequently, the final CAPRI design includes a web app with two interfaces (patient and health care professionals) and two NNs. The NNs provide regular follow-up via telephone or email to manage patients’ symptoms and toxicity, treatment compliance and care packages. Patients contact the NNs via a secure messaging system. Eighty clinical decision support tools enable NNs to prioritise and decide on the course of action to be taken.

**Conclusion:**

In our experience, the RPMS design process and, more generally, that of any complex intervention programme, is an important phase that requires a sound methodological basis. This study is also consistent with the notion that an RPMS is more than a technological innovation. This is indeed an organizational innovation, and principles identified during the design phase can help in the effective use of a RPMS (e.g. locating NNs if possible within the care organization; recruiting NNs with clinical and managerial skills; defining algorithms for clinical decision support tools for assessment, but also for patient decision and orientation).

## Background

The delivery of healthcare services for patients with chronic diseases requires more effective coordination between professionals and patients/relatives along the care pathway. In response, many health care organisations have implemented Remote Patient Monitoring Systems (RPMS). This type of intervention programme can improve patient follow-up and have a positive impact on the quality of care (reducing toxic effects, improving treatment compliance, limiting adverse events) and health-related costs (reducing the duplication of prescriptions and hospital readmissions) [1–5].

According to the Chronic Care Model [6, 7], an RPMS comprises three distinct components that facilitate the coordination of patient care: a) organisational methods (e.g. patient navigation program) [8, 9], b) e-health technology (e.g. web portal, apps) [10, 11] and c) patient engagement through information and training (e.g. health-literacy tools) [12, 13]. By combining one or more of these key components, many RPMS have been applied around the world in a variety of chronic diseases in recent years with some spectacular developments [14–16].

However, two recent literature reviews reported mixed results in terms of the effective impact of RPMS on quality of care and cost management [17, 18]. This is partly attributed to the fact that effective RPMS implementation remains a significant challenge. It depends on the processes accepted in relation to professionals and patients as well as on the local organizational context. It is important to understand the factors that can influence patient behaviour and healthcare practices, as well as the specific features of the intervention programme per se and its implementation context [5, 17, 19, 20]. The functional components of the RMPS must also be clearly described to facilitate implementation and replication. Hoffmann et al., for example, reported that only 39% of non-pharmacological interventions are adequately described. This lack of precision leads to replication difficulties in other settings [21]. Finally, it is obvious that the evaluation protocol must be designed *ex ante* to the intervention [22]. Otherwise, some data required may be omitted. Overall, these observations indicate a careful approach should be adopted when designing any RPMS. The more consideration given to the afore-mentioned issues during this design phase, the more effective the implementation is likely to be [20]. A precise design can reduce the risk of ineffective implementation, avoid additional re-design costs and prevent replication problems [19, 23].

In order to investigate the design process, an RPMS is defined as a complex intervention involving several interactions between various individuals, organisations and tools which in this case refer to the combination of stages outlined above [19, 24]. An in-depth literature review has been proposed to guide the development, implementation and evaluation of such complex interventions [19, 25–28]. One major reference is the UK Medical Research Council (MRC) that defines four stages comprising key functions and activities: *Development, Feasibility/piloting, Evaluation, and Implementation* [25, 29]. This widely used guideline is of particular interest for interventions involving e-Health technologies such as RPMS [30]. It helped us to use the intervention design process as the core development phase of this framework.

The aim of this paper is to report on our RPMS process design experience based on the CAPRI (Cancérologie Parcours Région Ile de France) Case Study. The RPMS, recognised as such by the French public authorities, consists of 2 NNs and technological support (a telephone platform and a web application). Its added-value has been assessed through a Randomized Clinical Trial (RCT) that significantly demonstrated an increased follow-up of the medical prescription, a reduction in the effects of severe toxicity, a decrease in hospital days, and a better patient experience in the follow-up of their disease [31]. The RPMS is the result of a design process carried out to improve the management of cancer patients throughout the care pathway. Cancer is an ideal field for developing RPMS since it is a major chronic disease (leading cause of death in addition to having significant social and economic consequences) [32, 33], and requires organizational innovations for improving coordination along the care pathway. We based our design research protocol on the MRC (Medical Research Council) framework [25, 29]. A series of research studies have been conducted in accordance with the MRC’s guidelines to identify the evidence base, develop the appropriate theory and to model process and outcomes during the development phase. The contributions are two-fold. Firstly, they provide insight into an oncology-specific RPMS and its design process, which is a current issue. Secondly, it contributes to the methodological debate regarding guidelines for developing complex interventions.

## Methods

### The implementation and project management team

The RPMS under test, namely, CAPRI (CAncerologie Parcours Région Ile de France), was trialled at the *Gustave Roussy* Institute (Villejuif, France), a leading European cancer centre with 449 beds and 94 day-care places, treating over 48,000 patients (year 2018).

The purpose of CAPRI was to develop a RPMS dedicated to patients receiving oral cancer medication - a treatment that has been used more extensively in recent years [34]. The use of such treatments is associated with organising and coordinating challenges [34–36]. Although most patients prefer oral therapy to IV therapy, they assume greater responsibility with oral therapies, and may encounter difficulties such as toxicity, which could affect treatment compliance. Furthermore, visits to health facilities are less frequent with primary care professionals potentially being the primary point of contact.

The initial stage of the project was devoted to the design of CAPRI (mid-2013-2015), and the second stage to implementation and evaluation (2016–2019). Three groups were formed depending on the work being carried out and the individual areas:

1. An expert group focused on the scientific aspects. Several disciplines were represented: oncologists, pharmacists, and health service researchers (management science and biostatistics);
2. A functional group was set up comprising hospital management personnel from various departments: Quality, Medical Information, Information System Management, Nursing Directorate, Outpatients, Pharmacy and Finance to make administrative decisions;
3. An operational working group including researchers from the expert group, and staff from different clinical departments to plan the daily agenda.

During the initial intervention design stage, the expert group met monthly, the functional group once every 3 months, and the last group on a weekly basis depending on project progression, in addition to telephone calls and emails.

The Principal Investigator of the evaluation programme was the Outpatient Senior Oncologist given his interaction with other healthcare professionals (MdP followed by OM). The Scientific Lead was a senior researcher in Healthcare Management Science (EM). A Programme Coordinator was also appointed to oversee all project management issues (MF).

### Methodological principles to support the RPMS design process

The design process was managed by the multidisciplinary team comprising the three groups. Their role was backed by various studies carried out at Gustave Roussy. Eight studies were finally outlined, based on the three principles covered in the MRC framework during the development phase [25]: (1) Identify the evidence base: to identify existing knowledge about similar interventions and the methods used to evaluate them, (2) Identify/develop an appropriate theory to promote the theoretical understanding of the likely process of change by drawing on existing evidence and theory and (3) Model process and outcomes prior to full-scale evaluation.

**Fig. 1:**
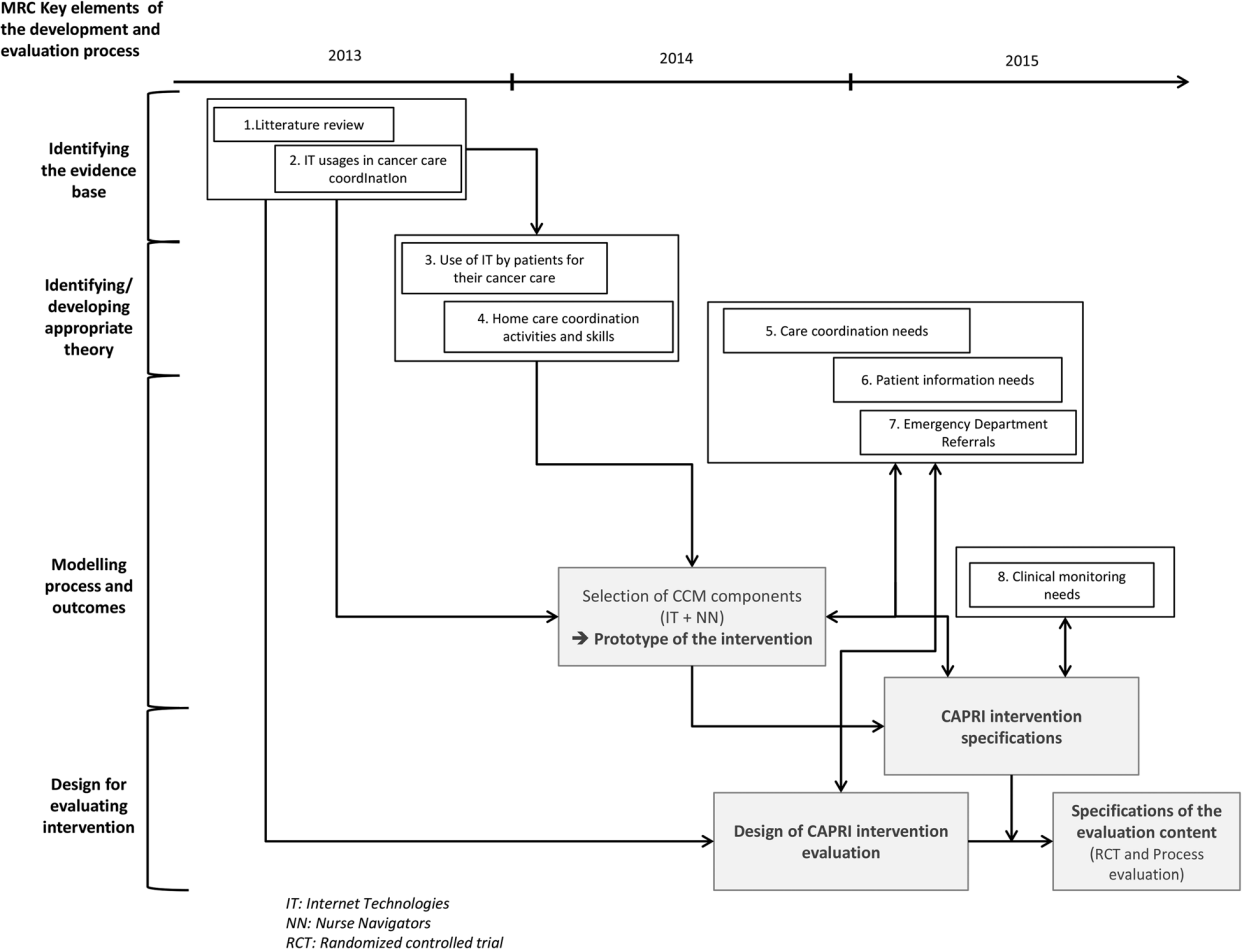
CAPRI design: overview of supporting research

The CNIL approved each study. As a result, several stages in the design process were carried out between 2013 and 2015 (see Fig. 1).

The various studies carried out in support of the CAPRI design process are also summarised in Table 1. Some studies have been published in more detail elsewhere [37–41]. The four exploratory studies (studies 1 to 4) provided a preliminary draft of the CAPRI design including selected components (health technologies and new organisational methods involving nurses specialising in coordination) and the main functions and interaction. The intervention proposal was then presented to potential stakeholders (study 5) to model the process and outcomes. A patient study was conducted simultaneously to identify the unmet information needs of cancer patients and understand the reasons behind patient dissatisfaction (study 6). A quantitative study on the appropriateness and potential avoidance of emergency visits was also carried out to assess the potential contribution of the intervention (study 7). Finally, interviews were held with Gustave Roussy professionals involved in patient follow-up (oncologists, support teams) to model patient monitoring and develop the necessary monitoring tools (protocols, clinical decision support tools) (study 8).

**Table 1:** Study methodology for designing CAPRI

N°	Studies	Objectives	Study design	Methods	Period
**1**	**Literature review** [37]	Select existing literature reviews to identify interventions to improve coordination on the cancer pathway	Literature review analysis	Selection of literature reviews via PubMed and extraction of interventions with a demonstrated effectiveness	2013
**2**	**IT usages in cancer care coordination**	- IT state-of-the-art in cancer care - Identify good practices to support efficient implementation	Literature review	Literature Review (pubMed and Cochrane Library) - Extraction of IT uses reported in the literature and classification - Recommended formulations	2013/2014
**3**	**Patient use of internet-based technologies** [38]	- Understand the current level of usage of internet-based technologies by patients - Assess their intention to use them for their health	Quantitative survey Gustave Roussy’s outpatient descriptive statistics and correlation analysis	Questionnaire-based survey carried out within seven outpatient departments over 7 days. - 3-part Questionnaire: (i) Use of internet through computers, mobile phones and tablets (ii) Willingness to use information technologies for health purposes (iii) Socio-demographics	2013/2014
**4**	**Home care coordination activities and skills** [39]	- Identify the need categories of patients and primary care providers for home care coordination - Quantify the volume of the activity generated by each category of needs.	Qualitative and quantitative analysis	Mixed method: (i) Qualitative phase: interviews with patients and focus groups with the NNs of the Coordinating Outpatient Care (COC) department at Gustave Roussy (ii) Quantitative phase: phone calls (made by both patients and primary care providers) received at the COC department. The caller, reason for the call and procedure performed were systematically reported and then analysed.	2014
**5**	**Care coordination needs**	- Understand operational care processes - Identify care coordination needs - Define how technological tools and nurses could prevent difficulties and facilitate care coordination between patients and professionals - Choose the location of NN	Interview survey	Interview survey with patients, hospital practitioners, primary healthcare providers and other hospital professionals	2014
**6**	**Patient information needs** [40]	Identify the unmet information needs of cancer patients and understand the reasons behind patient dissatisfaction	Interview survey and shadowing	Interviews with cancer patients attending a Meeting and Information Area (ERI) at Gustave Roussy and focus groups with ERI professionals. Data were analysed using vertical and horizontal open coding	2015
**7**	**Emergency Department referrals** [41]	Describe and quantify the appropriateness and potential avoidance of Emergency Department referrals	Electronic medical record review	Prospective review of the electronic medical charts of patients admitted in succession to the Emergency Department in August 2015. The appropriateness of referrals was assessed using a nationally validated classification system and local criteria. Potentially avoidable referrals were assessed using international classification systems and local criteria	2015
**8**	**Clinical monitoring needs**	- Identify clinical monitoring parameters - Define the monitoring guidelines	Interview survey	- NNs - Oncologists (referral physician according to site and supportive care)	2014/ 2015

## Results

The results are divided into two parts: the first part gives an insight into the RPMS design process whilst the second part describes the final Capri design.

### The RPMS design process

The main results of the studies conducted during this design phase are summarised in Table 2.

**Table 2:** Key findings from combined CAPRI design studies

N°	Studies	Sample	Main results	Principal findings for design intervention and implementation	Principal findings for evaluation
**1**	**Literature review**	3 literature review identified [42–44]	Effective intervention based on literature reviews: - Patient information - Decision –making aids for patients - Audiotaped consultation - Follow-up by nurses - Follow-up by GPs - Case management - One-stop clinic - Shared-care programme	Selection of components from the CCM	Combination of randomised controlled trial (RCT) and process evaluation
**2**	**IT usages in cancer care** **coordination**	46 articles analysed in realistic literature reviews	Identification of six uses of TIC: document management, dissemination of information and pooling of patient data, communication between stakeholders, aid in clinical decision-making, patient education and level of independence, personalisation and coordination of care pathway	- Definition of the functional basis provided by the technological tools used in the intervention programme - Implementation recommendation: promote the sharing of information and system integration, rigorously plan the design of the intervention, improve project management, work on tool ergonomics, plan the secure data strategy	Need to devise a robust evaluation strategy to assess the quality of life, satisfaction, organisational and economic impacts as well as the clinical outcome.
**3**	**Patient use of IT**	*n* = 1371 questionnaires (participation level = 85%) Median age of patients: 53.4 years, 70% were females	Access and use: 93% had home access to the Internet, 71% used a mobile phone every day and most patients reported never using tablets Willingness to use IT for their health: The most useful features: - Having access to electronic records, completion of a self-test to assess health status, communicating with physician via email, booking appointments and obtaining information about their disease - The least useful features: chatting with peer patients, communication via video Perceived ease of use: 84% confirmed that they were able to use a computer, tablet or smartphone	- Study provided cancer patients with an opportunity to use IT for health purposes, no major obstacles identified but the effects of age and socioeconomic status have to be addressed. - No need to equip the patient with any additional material (e.g. digital tablet). - Selection of priority functions to be integrated in the tool (data collection system, secure messaging system, information source provided, etc.). - Data security requirements - Key contacts	To be considered in process evaluation: Acceptance of the IT tool, patient profile with regard to IT, frequency of use and changes in use over time
**4**	**Home care coordination activities and skills**	Qualitative phase: 17 interviews with patients and 2 focus groups with NNs Quantitative phase: 543 phone calls received via COC platform	Five categories of NNs-related activities defined as: 1. Patient monitoring (e.g.: reporting side effects) 2. Navigation assistance (clinical pathways) 3. Managing technical problems (difficulties in drug or medical device delivery or equipment malfunction) 4. Explaining care protocols (e.g. clarification about the application of a drug prescription) 5. Collecting and transmitting patient data Although a significant proportion of the NNs’ activities involve patient monitoring (29%), most of the requirements (71%) relate to organisational issues.	- Definition of the NNs profile and development of the job description (role of case manager with clinical skills, knowledge of outpatient care and the healthcare system) - Need to develop tools for nurses to assist in the management of patient follow-up (clinical decision support, protocol)	To be considered in process evaluation: - organisational change triggered - Characteristics of NNs activities
**5**	**Care coordination needs**	59 individuals met, 45 interviews conducted −19 Patients −11 Community professionals(GP, Private nurses, Dietician, Pharmacist) - 29 Gustave Roussy Professionals	Potential benefits of the digital tool - Standardisation of follow-up informationProvision of practical information on a daily basis - Functions: long-distance consultation, document dispatch (results, evaluation, photos), monitoring of vital parameters, organisation of appointments, storage and permanent access to information, list of personal contacts Potential benefits of NNs - To answer telephone calls and receive alerts - To have explanatory consultations in addition to the normal reporting system (diagnosis, relapse, discontinuation of treatments, etc.) - To support patients along the pathway - To send information to various professionals involved in the patient’s treatment pathway (hospital and community) and to guarantee the link between patient, treating physician and referral oncologist NNs location: - Community professionals may have a lack of information and training in oncology, they have difficulties in having oncologist expertise	Warnings relating to the digital tool: - Does not replace direct or telephone contact - The information collected is not sufficient to trigger a decision and action - Avoids the risk of intrusion in the patient’s home Warnings regarding the role of the NNs – what the NN must not do: - Manage appointments, guarantee regulations and refer to emergency unit, responding to medical alerts and take decisions Conditions for a successful outcome: - Have a baseline (with clinical decision support tool) which is validated by all the committees, with warning thresholds and procedures to follow NNs’ profile: - Case manager role with clinical competencies, knowledge of the outpatient and care system - Ability to interact with the Hospital Information System NNs location: - Hospital: to have an easy access to the oncologist’s expertise	Population selection: patients treated with oral anticancer drugs RCT: - Choice of primary evaluation criterion/endpoint: Efficacy hypothesis: thanks to faster management of treatment-related side effects, patients participating in the CAPRI intervention programme will demonstrate a significant increase in Relative Dose Intensity (RDI) - Choice of secondary criteria: patient compliance, quality of life, patient experience, tumour response, Progression Free Survival, Overall Survival, toxic side effects and economic evaluation (medical and non-medical costs) Process evaluation: Study of changes in organisational transformations and, in particular, the impact of the intervention programme on the oncologists’ workload
**6**	**Patient information needs**	19 interviews with patients	- Patients were looking for treatment documentation on treatments but three types of non-medical information were also identified: a) Information on the care pathway, hospital and on health care system in general (e.g. administrative rules, departmental structure); b) Information on supportive care (e.g. services, activities) and how to contact professionals internally (within the hospital) and externally (e.g. dietician, psychologist); c) Information on living with cancer and its impact on daily activities. - Patient dissatisfaction is linked not only to the lack of medical information but also reflects other needs, which are not taken into account (e.g. expanding on information to make it understandable and useful).	- Information must be considered using an integrated and holistic approach to facilitate the patient navigation process and improve health-related literacy - Training of healthcare professionals is crucial, but this is not enough. The introduction of other, non-carer professionals is necessary to address a wide range of patient-related needs in a more effective and cost-efficient manner.	Assessment criteria used in the longitudinal analysis: Acceptance by patients
**7**	**Emergency Department (ED) referrals**	Electronic medical record review: 500 referrals related to 423 patients	- Referrals were appropriate in 61% of cases - Referrals were deemed potentially avoidable in 33.4% of cases, potentially avoidable in 14.4% and unavoidable in 52% of cases Opportunities to avoid referrals after index hospitalisation involved this hospital stay or discharge process in 66 cases (28%), the follow-up period in 59 cases (25%), or both in 66 cases (28%). Causes of potentially avoidable referrals may be linked to three main problems: - A lack of effective care during follow-up (lack of medical expertise, either on the part of the oncologist regarding chronic or intercurrent conditions or on the part of the GP about cancer) - Care coordination (lack of information for outpatient providers on referrals, and outpatient referrals omitted) - Patient management during the index hospitalisation (premature discharge or inadequate assessment of post- discharge risk)	- Lack of information from inpatient to outpatient providers but also vice-versa - Most inappropriate referrals needed consultations and not in a hospital setting - Merits of the GP to be in contact with the oncologist to improve the relevance of referrals - Need for tools to facilitate communication, legal framework development, financial incentives, training in shared medical management and patient education	Criteria regarding readmission and ED visits were added to protocol evaluation
**8**	**Clinical monitoring needs**	22 interviews with oncologists, NNs, and support packages	Drafting of clinical decision support tool in conjunction with a follow-up protocol through joint work between the NNs and the various Gustave Roussy Medical Discipline Leads regarding the information provided by analysing the medical and paramedical literature and obtaining expert opinions.	- Modelling of the follow-up process (initial NNs consultation, frequency of follow-up, items to be assessed, pooling of information) - Devising NNs follow-up tools (clinical decision support) - 80 validated clinical decision support tool	Design of NNs activities for improving evaluation criteria

They contributed in several stages to the design of the intervention programme and the assessment methods employed.

#### RPMS content

The literature reviews (studies 1 and 2, Table 2) taking into consideration the experiments carried out to coordinate care reported that a very wide range of tools and organisations are used to improve the care pathway. Some interventions are based on apps or tablets whereas others use telephone platforms. The role of case manager, coordination nurse or senior nursing clinician is often perceived as value added (studies 2 and 4, Table 2). As a result, the two main components retained, *e-health technologies* and *new organizational methods with the implementation of Nurse Navigators (NN)*, were deemed potentially appropriate to meet coordination needs identified in the specific case of CAPRI. Patient commitment, another important factor in the successful patient-professional relationship, is linked as much with IT usage as with the quality of the NNs relationship (studies 3 and 6, Table 2).

Each CAPRI component was also analysed in order to predict implantation problems. The introduction of IT with a patient survey (median age 53.4 years) [38], has allowed the most appropriate technological tool (web and mobile app with two interfaces: patients and outpatient professionals) to be identified alongside the priority features to be developed to make the technological tool attractive for patients (studies 3 and 5, Table 2). Similarly, the combined study on home care coordination activities has prompted a better understanding of the current practices and skills required for the NNs role (study 4, Table 2) [39]. Investigations have shown that NNs have to deal far more with organisational issues or routine activities in the life of a patient (e.g. how to travel with oral therapy) than with clinical ones.

Under the supervision of the three groups, these exploratory studies have led to a precise design, suggesting a technological tool, including a definition of these functions and a detailed description of the NNs role. The initial design could be discussed with potential users in order to *model process and outcomes* and define the major expected outcomes and the most relevant target population for the design of CAPRI.

#### Clinical decision support

The interviews carried out during study 5 (Table 2) highlighted the fact that NNs have clinical decision support tools to standardise patient follow-up in the very least (initial consultation, follow-up frequency and methods, evaluation parameters) to assess the situation, and finally to define the action to be taken. These tools were devised and validated with referral oncologists and health care support staff (study 8, Table 2), they were devised on the basis of NCI-CTCAE-V4 [45]. The questions to be put to the patients were defined in order to determine the severity of the recurring events, action (procedure to implement) and results (assessment of actions). These algorithms led to 3 action-based principles for NNs: advice given to patients, the organisation of a consultation (hospital or GP), and the organisation of a hospital admission. Ultimately, 80 clinical decision support tools have been developed allowing NNs to prioritise and define the action to be taken based on alert parameters. This study also highlighted that NNs should be located within the hospital where patients are treated in order to have easy access to the oncologist’s expertise if necessary.

### Evaluation protocol

Alongside the intervention design, one major point was to define the entire assessment system prior to the implementation process in order to outline the evaluation criteria, the data to be collected and the target population.

According to the literature [20, 46], the group of experts also adopted the concept of an evaluation process, which led to the definition of two evaluation methods for the design of the protocol to measure the impact of CAPRI [47]. Initially, as this is a type of study with a strong evidence base, a decision was taken to assess the « clinical » impact of the programme based on a randomised, controlled trial with a 700-patient cohort. The criteria for this study were determined using preliminary studies (studies 2 to 8, Table 2). The main evaluation criterion was defined as the relative dose intensity to assess compliance between the dose taken by the patient and the one scheduled in the protocol. Secondary endpoints were patient compliance with oral anticancer therapy, quality of life, patient experience, tumour response, Progression Free Survival (PFS), Overall Survival (OS) and the toxic side effects of treatment (severity and quantity). The RCT also includes an economic evaluation which adopts a societal perspective, assessing intervention, medical and non-medical costs. Finally, one endpoint focuses on the reason for emergency referrals. The results obtained in the Emergency Department study [41] have reinforced the value of the intervention programme in terms of reducing the number of unnecessary visits to the emergency department. Inappropriate and avoidable visits appear to be caused by inadequate referral to the most appropriate health professional, which is a key point in the follow-up process.

In addition, the difficulty in quantitatively measuring some of its effects and the behaviour of professionals in coordinating their actions, in particular, resulted in a longitudinal study. Collectively, and based on the analyses of articles in an attempt to RPMS evidence (studies 1, 2 and 8, Table 2), this approach was adopted to evaluate changes in the use and suitability of both tool and programme over time, focusing in addition on the behaviour of patients and professionals and how changes were implemented and knowledge was acquired during the implementation of the CAPRI programme.

### Legal issues

In addition, regulatory strategies specific to developing the evaluation of telemedicine/remote medicine systems in an experimental setting were required. Since the CAPRI follow-up system is based on a telemedicine activity (remote follow-up), a telemedicine contract had to be signed with the Agence Régionale de Santé Ile de France (ARSIF) (Parisian Regional Health Agency). This step took 7 months and the contract with the ARS Ile de France was signed in October 2015. A mandatory procedure was also required with regard to the Commission Nationale de l’Informatique et des Libertés (CNIL) to authorise the pooling of personal health-related information. The CNIL procedure was initiated only after signature of the ARS Ile contract, and was obtained in May 2016. These legal issues have governed the regulatory framework in which the CAPRI system can be used.

Apart from the timescales imposed by these regulatory authorities, compliance also impacted system design. For instance, the plan was to allow community professionals, and General Practitioners in particular, to document mutual patient information using the shared patient follow-up record. This option was abandoned since an agreement would have been required with each health care professional in accordance with regulatory requirements. This system was deemed to be too complex for routine use.

### A dynamic, iterative process

**Fig. 2:**
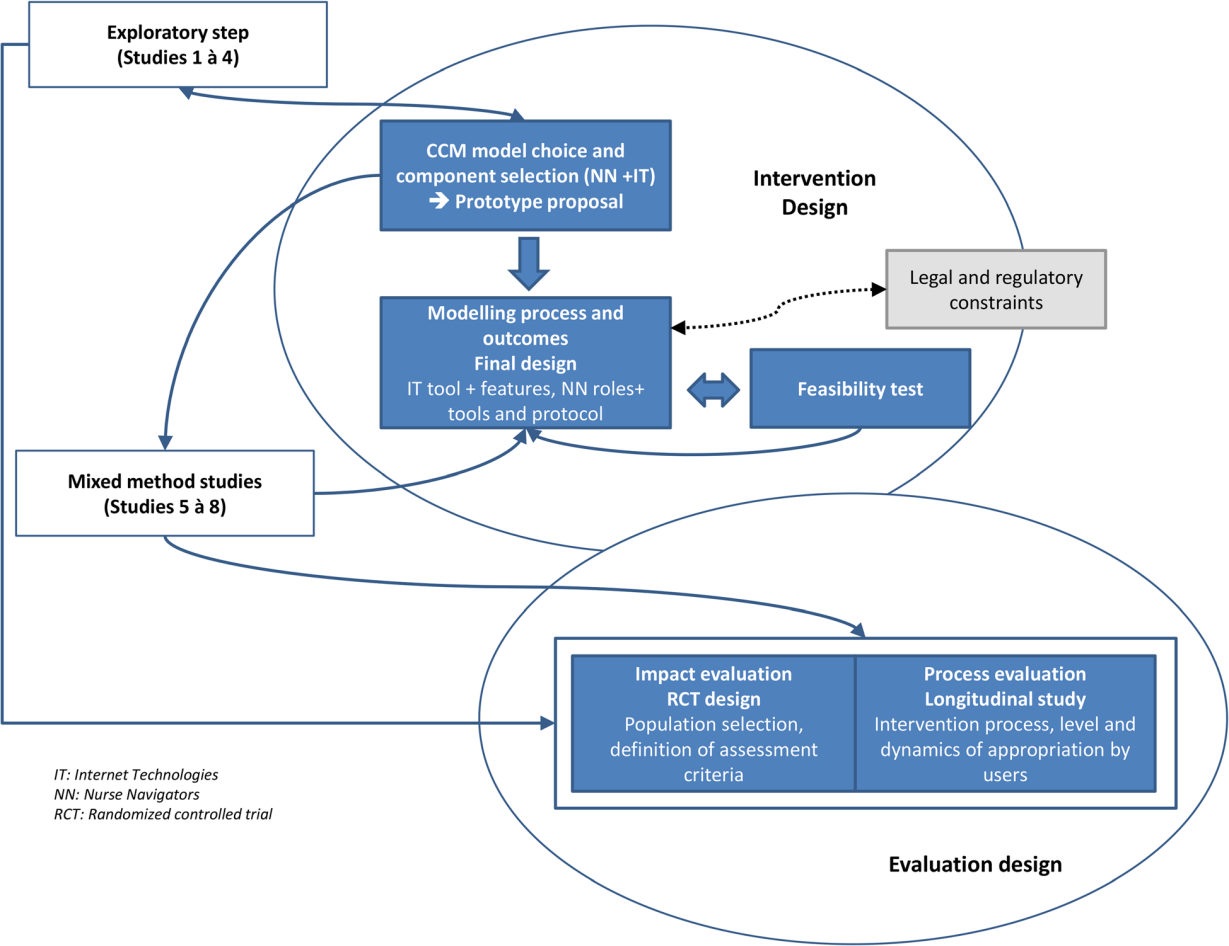
CAPRI design: an iterative process

All the issues outlined above refer to a dynamic design process. As summarised in Fig. 2, our design process allowed a simultaneous process to be carried out using an iterative approach towards the three MRC principles we applied.

This iterative approach allowed various obstacles to be identified (e.g. real and priority needs, local context specifics) and corrected prior to implementation (e.g. target intervention to the patients most likely to benefit; correct combination of NNs activities and e-health technology functions; holistic process design and outcome evaluation measure).

### Capri intervention specifications

**Fig. 3:**
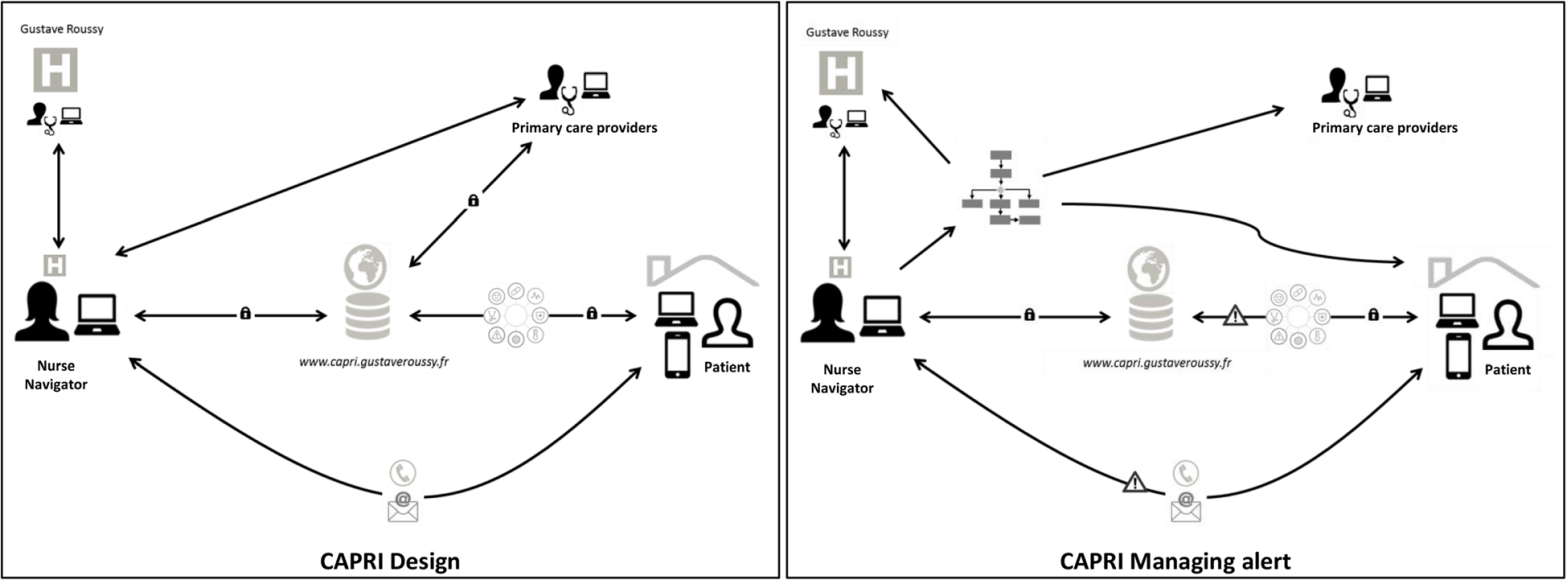
Final CAPRI design

The final CAPRI design includes a web/mobile app with two interfaces (patient and professional) and two Nurse Navigators (Fig. 3).

The organisational aspects are quite important. We have already described the different clinical decision support tools developed to assist NNs activities. The two NNs provide regular telephone follow-up to manage patients’ symptoms and toxicity issues, treatment compliance and supportive care needs. Patients have access to the app to record/track data, contact the NNs via a secure messaging system, view therapy and side effect information or store documents. The NNs are linked to health professionals involved in patient management.

#### Organisation of NNs activities

NNs conduct an initial assessment interview with each patient in-person or over the phone to identify his or her needs. The patient interview also includes a review of treatment, medical prescriptions and appointments. NNs then prepare the individual patient electronic medical record on the CAPRI application. Following this initial phase, NNs ensure patient follow-up (e.g. temperature, weight, pain, diet) remotely, through telephone interviews and emails, from Monday to Friday, during office hours only (from 9 am to 5 pm). Patients benefit from a regular phone follow-up in addition to individual contact depending on access difficulties, needs, and resources. In addition, NNs help patients to identify and overcome obstacles, provide health and practical information as well as emotional support, help patients to organise their appointments, help them to understand their conditions and treatments and help them to be actively involved in their care. They also forge links between the patient and hospital professionals and primary care providers (GP, private nurse, pharmacist, etc.) who are given access to the CAPRI application with the patient’s consent. To this end, NNs ensure that consultation reports, examination and test results and new medical prescriptions are available on the CAPRI application to all authorised healthcare providers at the time of an appointment. NNs inform health care providers about patient appointments, new treatments, new symptoms or difficulties, as required. Patient monitoring is described in a specific protocol prepared and validated by the expert group. During the initial assessment, each patient is given a starter box, which includes the following: login data to gain access to the portal, instructions for use and covering letters for healthcare providers, required (with information on the web portal, instructions on how to create an account and the, NNs’ contact details).

#### Web/mobile CAPRI application

The CAPRI application is available in web or mobile version and provides an interface to connect patients, hospital professionals, primary care providers and NNs.

The application offers patients several modules as shown in Table 3.

**Table 3:** Description of the main modules of the CAPRI patient application

Modules	Description
Messages	Secured messages to contact NNs
Follow-up	Tracking of follow-up measures (e.g. temperature, weight, pain, ingest) and if necessary, patient reporting of other symptoms
Appointments schedule	Display and save appointments on a personal schedule
Contact	Have access to an address book with contact details of professionals enrolled and other useful numbers
Information	Have access to reference websites providing information about the disease, the treatment and their side effects
Storage	Download, save and file documents relating to patient care (e.g. clinical and biological exams, patient medical records)
Reminders	Schedule reminders to take medications, arrange an appointment, plan exams, document personal measures

The CAPRI application provides NNs with a dashboard to enable them to monitor the individual electronic medical records of patients. Each time a patient is contacted, the NN can create intervention reports to record what they have done or discussed, and transmit the information to the professionals previously indicated by the patients. These professionals can log on the portal to communicate with the NNs online and access the relevant patient information. The system also generates automatic alerts which are sent to the patients or the NNs. The alerts and patients’ requests can be generated in different ways: 1) automatically, via the app, for instance while reporting follow-up measures (if the patient’s parameters are below or above predefined thresholds); 2) by the NNs during regular follow-ups; 3) by messaging/calling the patient or the professionals. The NNs assess the alert grade based on clinical decision support tools and determine the action to be taken according to navigation algorithms. Depending on the grade, the NNs can give advice, refer the patient to his or her primary care physician, or a *Gustave Roussy* professional or contact the relevant services in order to arrange hospitalisation or schedule an appointment for the patient.

## Discussion

This paper discusses the design of the CAPRI – an RPMS intended to improve the care pathway for cancer patients receiving oral medication. Two findings come to light.

Firstly, it outlines the crucial role of the design phase and provides an insight into the method required to carry out the process.

The way in which the system is accepted by patients and healthcare professionals as well as hospital managers is a key factor in effective implementation that starts during this design phase [48]. One major criterion was the considerable work carried out by the three groups focusing on identifying the coordination difficulties and priority needs in order to grasp a better understanding of the context and to define the main problem to be addressed by the intervention. Particular focus was given to the setting-up of these three groups (expert, functional and transversal working groups) and to incorporating individual contributions to provide an overall view of the intervention programme [49, 50]. The close collaboration between the three groups and within various disciplines and functions has allowed the programme to be designed in line with the real-life context, thus creating psychological ownership whilst better addressing the needs of patients, clinicians and managers. These are key factors in successful implementation [51, 52].

Our experience also shows that evaluation design must be carefully analysed in advance as a process evaluation. In terms of process and outcome evaluation measures, the work on identifying the target population, outcomes and programme content was crucial to improve the design, criteria and indicators to be followed in the randomised control trial, as noted elsewhere [53, 54] and in process evaluation. Another important point was to define a combined method comprising a randomised, controlled trial and a longitudinal qualitative approach which requires process evaluation. This process evaluation decision was taken in response to two objectives: to understand and describe how the system works in practice and to assess the level of suitability and retrace the dynamics of the latter in terms of patients and professionals alike. Indeed, both objectives are required in order to explain the results observed during the randomised study period [20, 46]. Thus it is a case of establishing a link over time between the dynamics of system suitability (acceptance method and sustainability/continuity) and changes in the results obtained within the randomised study context. Furthermore, in terms of the latter, the aim is two-fold. On the one hand, the randomised study seeks to highlight the contextual and behavioural factors that promote or hamper the implantation and continuity of co-ordination systems (the evaluation of the effects could not be detected to adequate extent by the randomised study and did not provide any justification, regardless of whether the effects were positive or negative). On the other hand, the longitudinal study highlights the key issues through a scientific approach, driving and optimising the implementation of coordination systems more effectively in line with local requirements.

These insights can contribute to the knowledge of development and implementation processes of healthcare intervention programmes. We based our research protocol on the MRC framework for the development and evaluation of complex interventions [25, 29]*.* The three principles of the development phase (i.e. “identify the evidence”, “develop an appropriate theory” and “model process and outcomes before a full scale evaluation”), were very helpful. In the experiments reported in the scientific literature, existing evidence in terms of intervention content, implementation strategy and process evaluation measures was sparse. Following the MCR principles, we collected evidence about the relevant target population, anticipated outcomes and the most suitable intervention. We developed the underlying theory and finally designed the intervention programme in operational terms, modelling the implementation strategy and the process and outcome evaluation measures. Indeed, the exploratory survey carried out initially and the literature review on care coordination requirements highlighted the priorities in terms of both the target population and outcomes. An intervention programme designed to address numerous requirements or several outcomes at the same time may produce confused and imprecise results. Similarly, these guidelines allowed the intervention content to be finely tuned following an iterative approach [25]. Although le MRC Framework proved useful in establishing the broad lines of the study, particular attention must be paid to the initial steps concerning the key elements in the « Development » stage. In fact, the method does not state this but, in our opinion, it is vital that interactions and repetitions are carried out between the « Developing appropriate theory », and « Modelling process and outcomes » phases described during this stage. The preliminary studies carried out during the development stage challenged the content of the intervention programme on many occasions and allowed us to define the interactions between the various components to align the evaluation criteria with the final actions taken within the intervention programme [55]. Furthermore, the regulatory and legal constraints seem an inherent part of the intervention design process when working on innovation programmes such as RPMS. In terms of our experience, their impact on the length of time to the implementation authorisation stage was significant and it seems vital to include this complex issue at the design stage [50].

Secondly, our experience shows the importance of organizational aspects in the RPMS context, precisely the coordination of care required for the organization of a remote follow-up of patients, as noticed elsewhere [56]. From a theoretical standpoint, many theories have already pointed out that the effectiveness of telemedicine can only be understood in its use, i.e. in social practices [57], or as a technology-in-practice in practice [58, 59]. In other words, the coordination of care cannot be predicted, and therefore, there is a limit to consider these organizational aspects during the design stage. In this context, this study provides elements on the aspects of care coordination that can be reasoned during the design phase. Our study shows that far from a definition of working rules of care coordination, some “principles” orienting its implementation, can be identified. The final CAPRI design provides the following principles that represent guidelines when designing coordination of care aspect into RPMSs:

-NNs have already proved their added-value in the patient management of different chronic diseases [14–16]. Here, we highlight that through their actions, NNs can enhance patient engagement in remote dialogue via the mobile app or telephone, by tailoring information, according to their needs [60], trigger clinical alerts, and develop coordination with the patient and other professionals (e.g. pharmacists, general practitioners, nurses). It requires a mix of clinical and managerial skills [61] that can lead to profile of NNs required.

-The clinical decision support tools provide a basis for defining the most appropriate answer for specific patient requirements as well as the most appropriate direction based on the clinical severity. Designing clinical decision support tools that are not only guides for evaluation, but also for orientation, whatever the organizational conditions to accomplish it, represent another recommendation.

-The need to link NNs to the technological application in order to process information more efficiently and direct patients accordingly suggests that there is a need to link the three factors of the Chronic Care Model [6, 7]: use of technological innovation (mobile app), the development of new coordination roles (NNs) and patient commitment. Furthermore, this choice prevent physicians from being overwhelmed by demands that are not related to their clinical expertise, thus allowing them to optimise their workload [62]. At the same time, it is important for NNs to be able to coordinate easily with medical oncologists. This has led to NNs located within the facility rather than on an external platform to limit the risk of distance or “physical boundary” [63]. Such a choice is also related to the hospital’s ability to recruit nurses, which depends on the number of patients followed.

-Finally, the selection of the feedback support, between the application allowing automated feedback and the phone, generally preferred by patients but more time-consuming for NNs, is another key point. In our design, we have integrated both options, the use of the RPMS showing the dominant use of the phone on the application afterwards [64, 65].

This study has various limitations. This was initially a specific case of RPMS, in a specific setting, namely Gustave Roussy, with the aim of monitoring patients receiving oral medication. All of these specific features require additional research in other settings, with other objectives, to define RPMS models. Secondly, the effective use of the RPMS and the study outcomes are not presented in the paper. The experimentation gave positive outcomes [31], and showed different insights about the use of the RPMS. As such, they suggest this type of design strategy can lead to positive outcomes, but it requires further evaluation to understand the relationships between the design phase, the implementation step, and the outcomes achieved. Thirdly, the strengths of our development process include the interactive approach combining the evidence base, theoretical framework and the involvement of a large network of stakeholders. However, this requires an important research investment in terms of time and money which is not always feasible. Fourthly, the use of the MRC framework meant that greater attention was focused on the context, resulting in the design of a customised intervention programme. Our aim was not to develop a design model that defines priority needs and the relevant content of such an intervention programme, but to show how to identify these needs and the key aspects in order to design the intervention. Moreover, by basing the design of the intervention programme on the local context, the risk is that the intervention designed may not be reproducible elsewhere. This is a key-aspect for large-scale circulation [66]. Future research should investigate this balance between designing a pilot study and the ability to transfer it.

## Conclusion

Despite limitations, our investigation reveals two findings about the RPMS content and the likelihood of encountering various issues relating to the implementation process during the design phase. Firstly, the RPMS programme is not only a technological innovation, something which is often outlined, but also an organisational innovation. This means that it is important to acknowledge the use of IT in conjunction with human practices and in a specific context (i.e. in our case, the 2 NNs and their relationship with other healthcare professionals). Secondly, this study confirms that the design phase of RPMS and, more generally, of any organisational intervention, must not be overlooked. As regards the methodological aspects of designing complex healthcare interventions, we wish to emphasise the fact that incorporation of the local context and relevant process evaluation are crucial in order to design an appropriate intervention programme and promote acceptance by users. Research programmes must therefore include the relevant dedicated stages. These preliminary phases warrant a constant review of the intervention content in order to ensure that it is fit for purpose in the given context. This should help to increase the likelihood of implementing an intervention programme in the most appropriate manner, which is a current issue in modern healthcare delivery systems.

## Data Availability

The data sets analysed in this study are available from the corresponding author on request.

## Abbreviations

CNIL: Commission Nationale de l’Informatique et des Libertés (CNIL) (French National Data Protection Commission)
ARS Ile de France: Agence Régionale de Santé (French Regional Health Agency) –Ile-de France
CAPRI: Cancérologie Parcours Région Ile de France (Regional Oncology Pathway)
MRC: UK Medical Research Council
NN: Nurse Navigators
RPMS: Remote Patient Monitoring System
